# Towards affordable biomarkers of frontotemporal dementia: A classification study via network’s information sharing

**DOI:** 10.1038/s41598-017-04204-8

**Published:** 2017-06-19

**Authors:** Martin Dottori, Lucas Sedeño, Miguel Martorell Caro, Florencia Alifano, Eugenia Hesse, Ezequiel Mikulan, Adolfo M. García, Amparo Ruiz-Tagle, Patricia Lillo, Andrea Slachevsky, Cecilia Serrano, Daniel Fraiman, Agustin Ibanez

**Affiliations:** 1 Laboratory of Experimental Psychology and Neuroscience (LPEN), Institute of Cognitive and Translational Neuroscience (INCYT), INECO Foundation, Favaloro University, Buenos Aires, Argentina; 2 National Scientific and Technical Research Council (CONICET), Buenos Aires, Argentina; 3 Instituto de Ingeniería Biomédica, Facultad de Ingeniería, Universidad de Buenos Aires, Argentina; 4 Faculty of Education, National University of Cuyo (UNCuyo), Mendoza, Argentina; 5 Centre for Advanced Research in Education, Periodista Jose Carrasco, Santiago, Chile; 6 Departamento de Neurologia Sur, Facultad de Medicina, Universidad de Chile, Santiago, Chile; 7 Gerosciences Center for Brain Health and Metabolism, Santiago, Chile; 8 Departamento de Neurociencia, Facultad de Medicina, Universidad de Chile, Santiago, Chile; 9 Physiopathology Department, ICBM and East Neuroscience Department, Faculty of Medicine, University of Chile, Santiago, Chile; 10 Cognitive Neurology and Dementia, Neurology Department, Hospital del Salvador, Santiago, Chile; 11 Centre for Advanced Research in Education, Santiago, Chile; 12 Servicio de Neurología, Departamento de Medicina, Clínica Alemana-Universidad del Desarrollo, Santiago, Chile; 13 Memory and Balance Clinic, Buenos Aires, Argentina; 14 Laboratorio de Investigación en Neurociencia, Universidad de San Andrés, Buenos Aires, Argentina; 15 Neuroscience Research Australia, Sydney, Australia and School of Medical Sciences, The University of New South Wales, Sydney, Australia; 16 Australian Research Council (ACR) Centre of Excellence in Cognition and its Disorders, Macquarie University, New South Wales, Australia; 17 Institute of Cognitive and Translational Neuroscience (INCYT), INECO Foundation, Favaloro University, Buenos Aires, Argentina; 18 Universidad Autónoma del Caribe, Barranquilla, Colombia; 19 Center for Social and Cognitive Neuroscience (CSCN), School of Psychology, Universidad Adolfo Ibáñez, Santiago de Chile, Chile

## Abstract

Developing effective and affordable biomarkers for dementias is critical given the difficulty to achieve early diagnosis. In this sense, electroencephalographic (EEG) methods offer promising alternatives due to their low cost, portability, and growing robustness. Here, we relied on EEG signals and a novel information-sharing method to study resting-state connectivity in patients with behavioral variant frontotemporal dementia (bvFTD) and controls. To evaluate the specificity of our results, we also tested Alzheimer’s disease (AD) patients. The classification power of the ensuing connectivity patterns was evaluated through a supervised classification algorithm (support vector machine). In addition, we compared the classification power yielded by (i) functional connectivity, (ii) relevant neuropsychological tests, and (iii) a combination of both. BvFTD patients exhibited a specific pattern of hypoconnectivity in mid-range frontotemporal links, which showed no alterations in AD patients. These functional connectivity alterations in bvFTD were replicated with a low-density EEG setting (20 electrodes). Moreover, while neuropsychological tests yielded acceptable discrimination between bvFTD and controls, the addition of connectivity results improved classification power. Finally, classification between bvFTD and AD patients was better when based on connectivity than on neuropsychological measures. Taken together, such findings underscore the relevance of EEG measures as potential biomarker signatures for clinical settings.

## Introduction

Behavioral variant frontotemporal dementia (bvFTD) is the second most common dementia in patients below age 65^1, 2^. Early diagnosis of this condition is difficult to achieve given its clinical overlap with other brain disorders^2^ and its variable pattern of brain atrophy at presentation^2, 3^. Developing effective neurocognitive biomarkers is thus critical to foster timely detection, track the impact of clinical and pharmacological interventions, and, more generally, alleviate associated financial burdens^4^. Previous research, mainly rooted in imaging techniques, has pointed to functional connectivity (FC) alterations as biomarker candidates for bvFTD^5–7^. However, the high costs and restricted availability of neuroimaging equipment clash against the need to establish affordable and massive markers for neurodegenerative diseases^5, 6, 8^. Instead, electroencephalographic (EEG) tools have emerged as a promising alternative due to their low cost, greater accessibility, and growing robustness^5, 8^. Against this background, the present study aimed to assess the sensitivity and specificity of EEG-derived FC patterns as potential biomarkers of bvFTD.

Previous resting-state EEG studies have pursued similar aims relying on visual EEG rating scales and power spectral measures. Results remain inconclusive, as some studies found significant differences between bvFTD patients and controls^9, 10^, others found inconsistent differences across disease stages^11^, and some found no significant results at all^12–14^. Findings are also inconsistent across the few electromagnetic studies employing connectivity methods. For instance, relative to controls, bvFTD patients exhibited increased phase lag index in the delta band (with regional connectivity differences emerging only in single-electrode analyses), frontal alterations in alpha band, and no differences in beta bands^15^. Also, Hughes and Rowe^16^ have shown reductions in frontotemporal beta coherence during an active task, but they did not perform resting-state analysis and did not include a second patient group to capture the specificity of the measure. Another study^17^ showed increased degree in the alpha band for bvFTD, although no topographical analysis was reported. However, an investigation analyzing connectivity via synchronization likelihood found no differences between bvFTD and controls^13^. In sum, no clear picture emerges from the available literature, arguably due to the use of various metric properties, the inconsistent parameters employed to assess regional or global connectivity, and the paucity of studies including contrastive patient groups.

To contribute to the quest of sensitive connectivity biomarkers of bvFTD, here we used a promising new connectivity measure called Weighted Symbolic Mutual Information (wSMI)^18^. In particular, this method proves more sensitive than previous approaches to EEG connectivity (phase-locking value, phase-lag index, and power spectral densities) used to evaluate network abnormalities in other pathological samples^18^. Moreover, wSMI has already proven sensitive to detect aberrant networks in other neurodegenerative conditions^19^. Instead of measuring basic oscillatory correlations, wSMI assesses the non-linear coupling of information sharing among distant networks. It presents several advantages, including a fast and robust estimation of the signals’ entropies, the detection of nonlinear coupling, and the absence of spurious correlations between EEG signals arising from common sources^18, 20^. In this study, our analysis focused on the alpha and beta bands, which are systematically affected in bvFTD^10, 21–23^. In addition, to evaluate the specificity of our results, we included a group of Alzheimer’s disease (AD) patients, who feature a partially contrastive clinical and neurodegenerative profile^5, 6^. Finally, to assess the relevance of wSMI-based connectivity as a potential biomarker, we implemented two strategies: first, we used such data to automatically classify patients and controls via a support vector machine (SVM); second, we compared the classification power yielded by connectivity patterns with those obtained through neuropsychological measures and through a combination of both approaches.

Given that bvFTD patients are crucially characterized by frontotemporal atrophy^24, 25^, we hypothesized that they would exhibit reduced information sharing across frontotemporal hubs relative to both other groups. To achieve a comprehensive assessment of information sharing in each group, we assessed various dimensions of connectivity, namely, average connectivity, connectivity as function of distance, and seed analysis. Also, based on previous imaging studies^24^, we anticipated that neuropsychological tests (specifically designed to identify clinically impaired subjects) would prove more sensitive than connectivity measures to discriminate between patients and controls. Yet, we predicted that the classification accuracy yielded by neuropsychological tests would be boosted when complemented by EEG connectivity data. Finally, regarding discrimination between patients (bvFTD vs AD), we expected that specific patterns of aberrant connectivity would provide a better classification rate than neuropsychological tests.

## Materials and Methods

### Participants

The study comprised 52 subjects from an ongoing multicenter project^7, 24, 26–30^. Our target group consisted of 13 patients fulfilling revised criteria for probable bvFTD. The control group included 25 subjects matched in sex, age, and years of education. Also, to test the specificity of potential connectivity alterations in bvFTD, we recruited 13 AD patients who fulfilled the international NINCDS-ADRDA criteria. All patients were in initial disease stages. Therefore, since bvFTD and AD differ in their typical age of symptom onset and initial diagnosis^2^, the latter sample was older than the former. Accordingly, analysis of the AD group was based on comparisons with its own sex-, age-, and education-matched control group (*n* = 18) (Table 1). For more details, see Supplementary Data 1. All subjects signed an informed consent and all experimental protocols were performed in accordance with relevant guidelines and regulations of the Declaration of Helsinki. This study was approved by the Institutional Committee of Ethics of the involved institutions. Finally, we performed an estimation analysis of the sample size of our main comparison (bvFTD vs. controls). Results showed that the number of participants in each group conferred sufficient statistical power to reach reliable effects (see Supplementary Data 2).

**Table 1:** Demographic and neuropsychological results.

	bvFTD patients	Controls matched with bvFTD patients	*p*-value	AD patients	Controls matched with AD patients	*p*-value
Sex (female:male)	13 (7:6)	25 (15:10)	0.71	13 (11:2)	18 (12:6)	0.26
Age (years)	69.31 (10.55)	70.40 (5.22)	0.67	75.62 (9.42)	72.28 (4.42)	0.20
Education (years)	15 (3.34)	16.96 (3.47)	0.10	12.77 (7.60)	15.94 (3.35)	0.12
ACE (global score)	70.77 (10.66)	92.17 (6.84)	<0.001	78.62 (11.86)	93.12 (6.06)	<0.001
RAVLT (immediate recall score)	24.23 (8.87)	42.89 (10.07)	<0.001	27.46 (8.25)	42.68 (10.19)	<0.001
RAVLT (delayed recall score)	3.69 (2.81)	7.28 (3.32)	0.002	1.31 (3.12)	7.11 (3.38)	<0.001
IFS (global score)	15.65 (4.39)	24.94 (2.25)	<0.001	17.77 (7.57)	25.40 (2.26)	<0.001

Means and (standard deviation). bvFTD = behavioral variant of frontotemporal dementia, AD = Alzheimer disease. We used t-test for variables comparisons between groups and, particularly, the pearson chi squared test for sex variable.

### Neuropsychological tests

All participants completed three neuropsychological tests tapping different domains: (i) the INECO Frontal Screening battery^31^, a sensitive tool to detect executive dysfunction in demented patients; (ii) the Rey Auditory Verbal Learning Test (RAVLT), which evaluates is sensitive to memory and learning impairments in neurodegenerative conditions^32^; and (iii) the Addenbrooke’s Cognitive Examination, a widely used tool for early detection of dementia. For more details about the tests, see Supplementary Data 3. The patients samples’ scores in each test and statistical comparisons with controls are offered in Table 1.

### EEG

#### EEG recordings and preprocessing

We recorded resting-state high-density electroencephalography (EEG) signals during a ten-minute resting-state protocol^33–35^ using a Biosemi Active-two 128-channel system. The protocol’s duration guaranteed at least five minutes of artifact-free signal per subject. The signal was sampled at 1.024 Hz and referenced to linked mastoids. Preprocessing was implemented following standard protocol (see Supplementary Data 4 for details).

#### Functional connectivity analysis

Functional connectivity between electrodes for each subject was computed through the wSMI measure, which provides a non-linear index of information sharing between two signals^18^. The analysis followed the same protocol reported in previous works of our group^19, 20^ (see Supplementary Data 5 for details).

To analyze specific topographic patterns of connectivity, we implemented three complementary and sensitive analyses, based on defined regions of interest (ROIs) covering the whole scalp, that have been widely used in previous research^27, 36, 37^. First, to assess the strength of association between ROIs, we calculated their average connectivity^37^. We focused on the interaction between all ROIs of the scalp to avoid any bias resulting from the selection of a priori regions associated with the fronto-temporal pattern of atrophy in bvFTD^1, 27, 38^. Thus, we were able to evaluate whether our hypoconnectivity hypothesis was constrained to specific regions or widely distributed over the scalp without any a priori bias. Second, to evaluate the extent to which connectivity between ROI-internal and ROI-external electrodes relied on local, mid-range, and long-range connections, we calculated their connectivity as a function of distance^27, 36, 37^. As in previous studies with neurodegenerative diseases^27, 36^, this analysis indicates whether connectivity strength is differentially affected in patients relative to controls according to the distance between connections. Finally, to identify the connectivity pattern of the regions yielding the greatest between-group differences in the previous analyses, we subjected them to seed analysis^37^, which is commonly used in neurodegenerative research.

**Figure 1:**
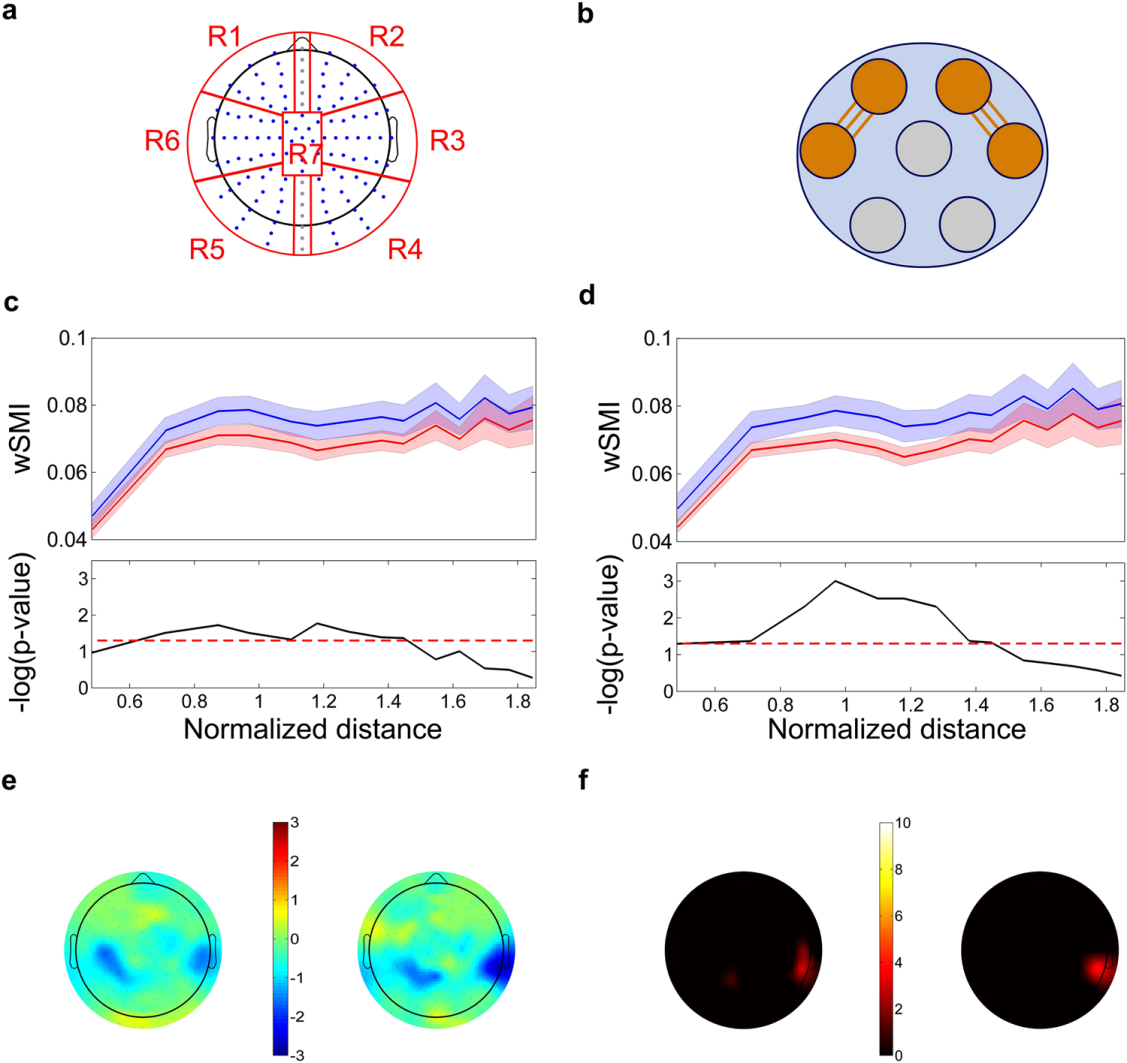
Functional connectivity analysis. (**A**) ROIs defined to analyze specific topographic connectivity: left frontal (R1), right frontal (R2), right temporal (R3), right posterior (R4), left posterior (R5), left temporal (R6), and central (R7). (**B**) Average connectivity between regions: significant differences for the average connectivity were found between the right frontal and right temporal ROIS (bvFTD: *M* = 0.06, *SD* < 0.01; controls: *M* = 0.07, *SD* = 0.01), and between the left frontal and the left parietal ROIs (bvFTD: *M* = 0.06, *SD* < 0.01; controls: *M = *0.07, *SD* < 0.01). (**C**) Connectivity as function of distance of the left frontal ROI: bvFTD patients (red) and controls (blue). Results are shown as -log (*p*-value) by distance; *p*-values crossing the dotted line are < 0.05. (**D**) Connectivity as function of distance of the right frontal ROI: bvFTD patients (red) and controls (blue). Results are shown as -log (*p*-value) by distance; *p*-values crossing the dotted line are < 0.05. (**E**) Seed analysis (median values): scalp maps of the median value of *p*-values (from Wilcoxon test between bvFTD patients and controls) are shown for the left frontal (left) and right frontal (right) seeds. The color bar indicates -log [median (*p*-values)] times the sign of W, where W is the Wilcoxon statistics minus the expected value under the null hypothesis. Values > 1.3 or < −1.3 are statistically significant. (**F**) Seed analysis (FDR correction): scalp maps quantifying the number of connections (associated to the seed ROI) yielding differences (*p*-value from Wilcoxon test between bvFTD patients and controls with FDR < 0.05) for each electrode. The maps show the results for the left frontal (left) and the right frontal (right) seeds. The color bar indicates the number of connections with statistically significant differences (*p*-values < 0.05).

*Average connectivity between ROIs*: First, we assessed average connectivity between ROIs to identify altered connections in patients compared to controls. ROIs were defined in seven standard regions: left frontal, right frontal, left temporal, right temporal, left posterior, right posterior, and central (Fig. 1A). To quantify the strength of between-ROI connections, we estimated the averaged connectivity values of all inter-electrode connections linking any two ROIs. This was repeated for all the possible between-ROI connections, resulting in 21 values. To compare these measures between groups, we applied a Wilcoxon test corrected with false discovery rate (FDR) to address the multiple comparisons problem.

*Connectivity as function of distance*: By assessing connectivity as a function of distance we evaluated whether local, mid- or long-range connectivity was affected in the patient samples, relative to controls. These distance ranges were defined by dividing the total distance range in three equivalent parts: short range (less than 0.6), mid-range (between 0.6 and 1.2), and long-range (more than 1.2). For details about the underlying statistical analysis, see Supplementary Data 6.

Comparisons between the results of patients and controls were based on a Montecarlo permutation test with bootstrapping^39^, as done in previous works^36^. This method overcomes problems due to multiple comparisons and assumptions on data distribution^40^. The number of randomly simulations partitioning the data (permutations) was set to 5,000. A *p*-value was thus obtained for each distance, but only those below.05 were considered significant.

*Seed analysis*: Finally, each electrode from the ROIs yielding the greatest differences between bvFTD patients and controls in our previous analyses (average connectivity and connectivity as a function of distance) was framed as a seed (henceforth called ROI-internal electrodes), and its connectivity was analyzed relative to every electrode outside these ROIs (henceforth termed ROI-external electrode) on the scalp via a Wilcoxon test. Thus, each connection between any ROI-internal electrode and any ROI-external electrode was associated to its own *p*-value –i.e., every ROI-external electrode is associated to as many *p*-values as ROI-internal electrodes selected as seeds. Then, we applied two strategies to analyze the ensuing *p*-values. First, to better characterize the topography of connectivity differences between groups, we computed the median score of the *p*-values corresponding to each ROI-external electrode. This strategy allows quantifying the differences for all electrodes relative to a ROI across the scalp, thus revealing which specific set of electrodes accounts for the major differences in that ROI. Second, the original *p*-values were recalculated via the FDR method to evaluate which differences proved most robust.

**Figure 2:**
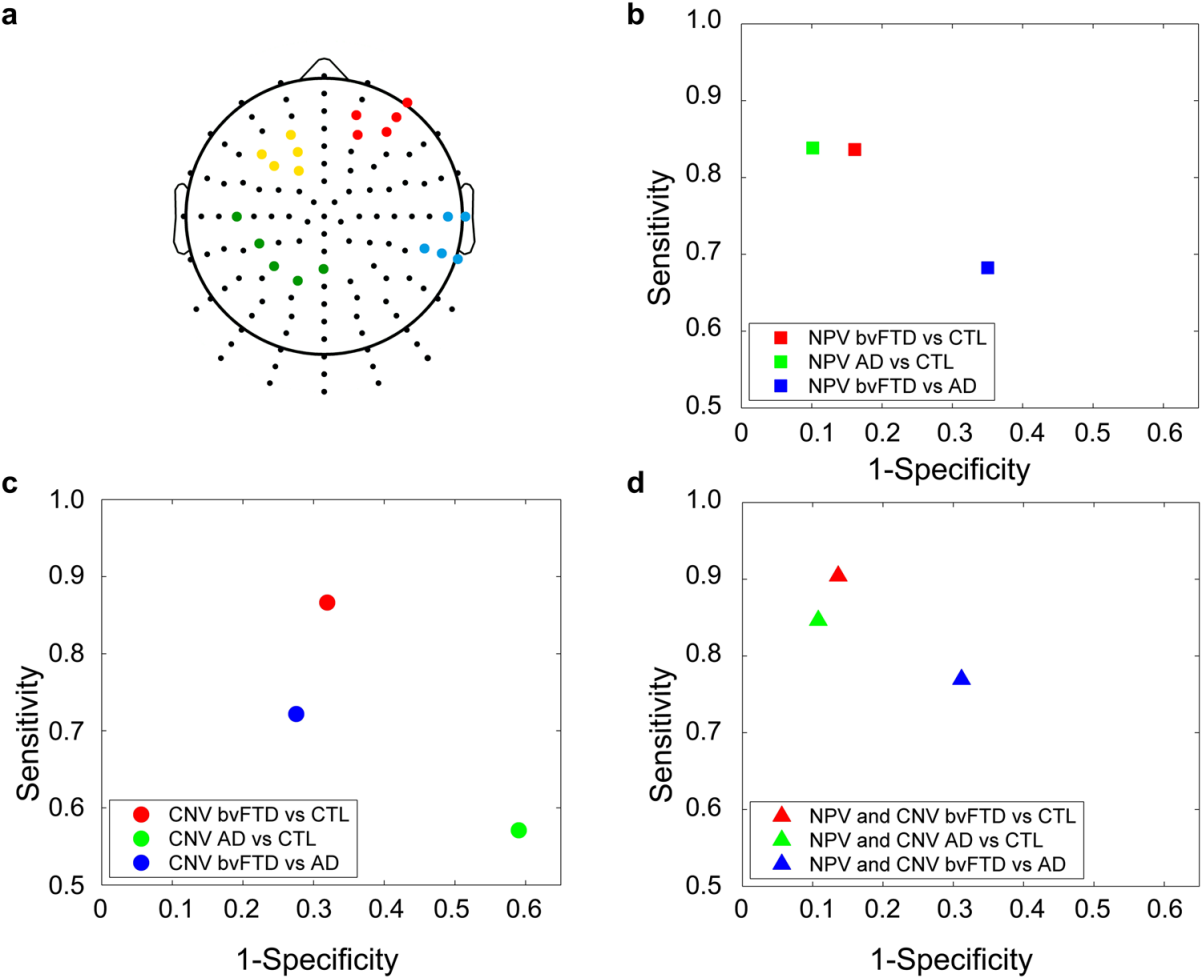
Classification analysis. (**A**) ROIs used for classification analyses. ROIs were defined are based on the major differences found in the seed analysis for the bvFTD comparison respect to controls: the light-blue and green one are the results from the first seed analysis (where we used the Frontal Left and Frontal Right seed from Fig. 1A), while the yellow and red one are the results from the second seed analysis, in which we used the light-blue and green ROIs from the first analysis as seeds. The average connectivity of these ROIs was used to define four of the CNV indexes for the classification analysis. (**B**) Classification analysis based on the NPS variables. The classification rates obtained were 83,8% for bvFTD vs Controls, 88,3% for AD vs Controls and 66,7% for bvFTD vs AD. (**c**) Classification analysis based on the CNV variables. The classification rates obtained were 72,7% for bvFTD vs Controls, 44,9% for AD vs Controls and 72,2% for bvFTD vs AD. (**D**) Classification analysis based on the combination of CNV and NPS variables. The classification rates obtained were 87,4% for bvFTD vs Controls, 88,0% for AD vs Controls and 72,9% for bvFTD vs AD.

The first seed analysis revealed which connections between pairs of ROI-internal and ROI-external electrodes differed significantly between groups (based on the median of *p*-values, see Fig. 1E). Then, the ROI-external electrodes involved in such connections were used as new seeds (see Fig. 2A). To identify the latter, we first averaged the results from the seed analysis of the left frontal and right frontal ROIs, for each ROI-external electrode. Then, we selected from each region with significant results (see Fig. 1E) the five ROI-external electrodes showing the highest differences between samples. These new seeds were used to identify a new set of ROI-external electrodes showing significant differences between bvFTD patients and controls. The aim of this second seed analysis was to specify which electrodes support the differences between groups across the scalp. The results from the first and second seed analyses were then used to estimate values for a classification analysis (see Classification analysis section).

### Disease control group

To test the specificity of our results, we also assessed AD patients with the three complementary approaches described above. First, we tested whether the alterations we expected to find in bvFTD patients were specific to them or shared with a contrastive form of neurodegeneration. To this end, connectivity patterns in the AD sample were compared to those of its corresponding control group (see Table 1). Then, such patterns were considered alongside neuropsychological test scores as predictors in a series of classification analyses (see Classification analysis section).

### Classification analysis

To assess the relevance of our EEG-derived results, we performed classification analyses considering three sources of information: (i) neuropsychological variables (NPVs) alone, (ii) connectivity variables (CNVs) alone, and (iii) a combination of both. This allowed us to determine the best index classifying between bvFTD patients and controls.

A first classification model was implemented considering only the global scores and the subscores of each neuropsychological test as predictor variables (see Supplementary Table 1 for a list of the 19 NPV variables). Then, we tested a second model in which only the connectivity differences between bvFTD and controls were framed as predictors. For this, we defined six indexes (calculated for each subject) to evaluate the robustness of functional connectivity. The first two indexes were estimated from the ROIs yielding the greatest between-group differences in the distance analyses (left frontal and right frontal). These analyses were based on mean connectivity calculations for the distance ranges with significant correlations (0–1.45 for left frontal and 0.6–1.45 for right frontal). The other four indexes were the average connectivity between the four pairs of ROIs that emerged as results from the second seed analysis: each of the new frontal ROIs (left and right) together with the left parietal and right temporal ROIs (see Results section and Fig. 2A). Thus, six CNVs were associated to each subject to evaluate their classification capacity: (i) left frontal connectivity distance, (ii) right frontal connectivity distance, (iii) connectivity between the right frontal seed and right temporal hubs, (iv) connectivity between right frontal seed and left parietal hub, (v) connectivity between the left frontal seed and right temporal hubs, and (vi) connectivity between the left frontal seed and left parietal hubs. Note that the first two CNVs were related to connectivity as a function of distance whereas the remaining four emerged from the seed analysis (see Supplementary Table 1). Finally, we analyzed a third model that combined all NPVs and CVNs as predictors.

A separate SVM was implemented for each of the above models: (i) NPVs alone, (ii) CNVs alone, and (iii) a combination of NPVs and CNVs (algorithm details are shown in Supplementary Data 7). Then, we tested each model’s precision in classifying between (a) bvFTD patients and controls, (b) AD patients and controls, and (c) bvFTD patients and AD patients.

The SVM algorithm involved a training phase and a classification phase. In the training phase, a subset of the data (corresponding to seven randomly selected subjects from each group) and their corresponding classes (subject condition) were used to determine the classification parameters (for details, see Supplementary Data 8). Then, in the classification phase, the rest of the data were segregated as one of two classes. This procedure was repeated 1,000 times and yielded mean sensitivity and specificity values together with their corresponding standard deviations (see Results section). The sensitivity and specificity values for each group of variables were compared using a Hotelling’s T-square test, which compares a pair of variables for two independent samples.

## Results

### Functional connectivity results

#### Average connectivity between regions

Compared to controls, bvFTD patients presented reduced information sharing between left frontal and temporal ROIs (*p* = 0.04), and between right frontal and temporal ROIs (*p* = 0.02) (Fig. 1B), with large effect size as shown by Cohen’s *d* index: 0.98 and 1.11, respectively. No significant connectivity differences emerged between the other ROIs (see Supplementary Table 2).

#### Connectivity as a function of distance

The analysis of connectivity as a function of distance revealed hypoconnectivity for bvFTD patients relative to controls, particularly in frontals ROIs. For the right frontal ROI, although significant differences emerged in the short-distance range (0–0.6), the largest significant differences involved mid and long ranges (from 0.6 to 1.4, see Fig. 1D). Likewise, for the left frontal ROI, the principal differences appeared in mid and long ranges (from 0.6–1.40, see Fig. 1C). These significant results reach larges effects sizes (Cohen’s *d* > 0.90 and Cohen’s *d* > 0.80 for effects related to each frontal ROI, respectively; see Supplementary Figure 1). No significant differences emerged in any other ROI.

#### Seed analysis

We conducted seed analyses in the bvFTD sample considering the ROIs yielding consistent connectivity differences, namely, the left and right frontal ROIs. The ROI-internal electrodes from these regions exhibited decreased connectivity with the ROI-external electrodes on left parietal and right temporal regions between patients and controls (Fig. 1E illustrates the median score of the p-values corresponding to each ROI-external electrode). Connections between the latter regions and frontal hubs were particularly robust, as they showed a large effect size (mean Cohen’s *d* value of 0.98 and 0.88 for the left parietal and right temporal regions, respectively) and were the only ones which survived correction via FDR (Fig. 1F).

We then conducted a second seed analysis focused on the regions yielding differences between bvFTD patients and controls (left parietal and right temporal regions) (Fig. 1E). To identify these new seeds, we first averaged the results from the seed analysis of the left frontal and right frontal ROIs, for each ROI-external electrode of the left parietal and right temporal regions (Fig. 1E). Then, for each of these regions, we selected the five ROI-external electrodes showing the highest differences between samples (showed in the Fig. 2A., green dots for the ROI-external electrodes from the left parietal region, and light blue for the right temporal one). Following the same procedures employed in our first analysis, we used these new seeds to identify a new set of ROI-external electrodes showing significant differences between bvFTD and controls (considering median values of Wilcoxon Tests at *p* < 0.05) (Fig. 2A). These new significant ROI-external electrodes were framed as two new ROIs for subsequent analysis: new left frontal and new right frontal.

The electrodes from these four regions identified in both seed analyses (the left parietal and right temporal in the first, and the new left and right frontal in the second one) were then used to calculate connectivity predictors in the classification analysis. To estimate these predictors for each subject, we first calculated the mean connectivity between the electrodes from the left parietal region and the two set of electrodes from the new frontal right and left regions, separately. The same was done between the right temporal region and the two frontal ones. These fronto-parietal and fronto-temporal connections were considered given the connectivity alterations observed in bvFTD from our previous analysis.

**Figure 3:**
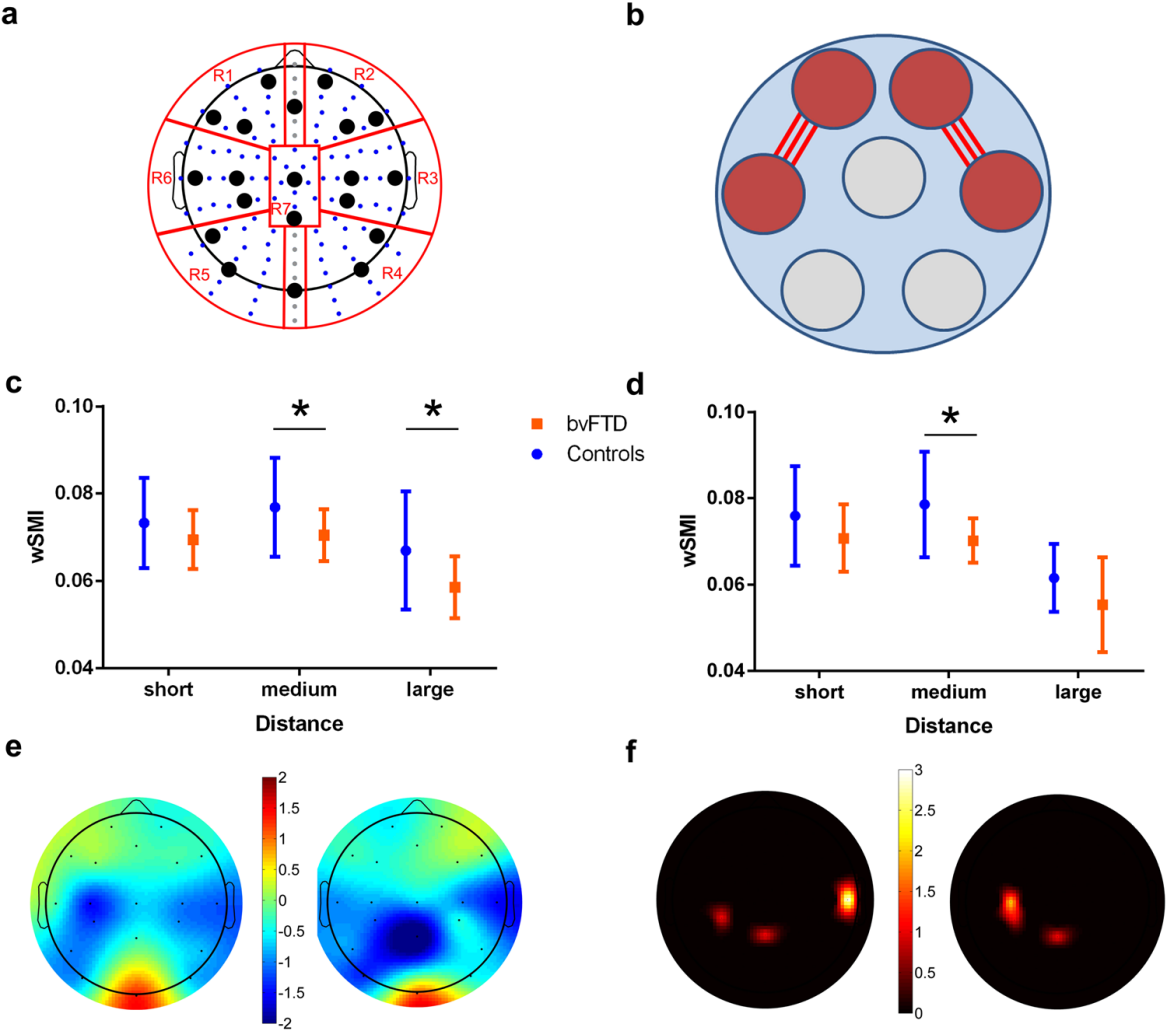
Functional connectivity analysis for a subset of 20 electrodes. (**A**) ROIs defined to analyze specific topographic connectivity: left frontal (R1), right frontal (R2), right temporal (R3), right posterior (R4), left posterior (R5), left temporal (R6), and central (R7). Marked electrodes in black are subset of 20 electrodes used in these analyses. (**B**) Average connectivity between regions: significant differences in average connectivity were found between the right frontal and right temporal ROIs (bvFTD: *M* = 0.065, *SD* < 0.01; controls: *M* = 0.075, *SD* = 0.01; *p-*value = 0.05,), and between the left frontal and the left parietal ROIs (bvFTD: *M* = 0.065, *SD* < 0.01; controls: *M* = 0.072, *SD* < 0.01; *p-*value = 0.05). (**C**) Connectivity as a function of distance of the left frontal ROI. Mean connectivity was calculated for the three distance ranges (short = 0.5–0.8, medium = 0.8–1.4, long = 1.4–1.8). Using permutation tests with 1000 iterations, we found significant differences for the medium (*p*-value = 0.04) and long (*p*-value = 0.02) ranges. (**D**) Connectivity as a function of distance of the right frontal ROI. Mean connectivity was calculated for the three distance ranges (short = 0.5–0.8, medium = 0.8–1.4, long = 1.4–1.8). Using permutation tests with 1000 iterations, we found significant differences for medium range connections (*p*-value = 0.01). (**E**) Seed analysis (median values): scalp maps of the median value of *p*-values (from Wilcoxon tests between bvFTD patients and controls) are shown for the left frontal (left) and right frontal (right) seeds. The color bar indicates -log [median (*p*-values)] times the sign of W, where W is the Wilcoxon statistics minus the expected value under the null hypothesis. Values > 1.3 or < −1.3 are statistically significant. (**F**) Seed analysis (FDR correction): scalp maps quantifying the number of connections (associated to the seed ROI) yielding differences (*p*-value from Wilcoxon tests between bvFTD patients and controls with FDR < 0.05) for each electrode. The maps show the results for the left frontal (left) and the right frontal (right) seeds. The color bar indicates the number of connections with statistically significant differences (*p*-values < 0.05).

Replication *in a low density EEG setting*: Compared to other neuroimaging tools (such as fMRI, or MEG), EEG offers low cost and higher portability, which paves the way for massive application. However, this applies only partially to high-density equipment as the one used in this research, which makes it necessary to assess whether similar results could be obtained in low-density acquisition settings. To this end, and following a previous study^35^, we repeated our three analyses considering only a set of 20 electrodes from the 10/20 system (as used by some portable EEG equipment^41–43^). The ensuing results replicated the patterns reported in our main study: of frontal hypoconnectivity, reduction of connectivity strength in medium and large distances, and abnormal connections involving fronto-temporal and fronto parietal regions of the scalp (See Fig. 3).

### Disease control group

We also examined functional connectivity differences between bvFTD and AD patients. We considered the two ROIs that yielded the more consistent differences between bvFTD and controls: the left and right frontal ROIs. Average connectivity was similar between frontal ROIs (Supplementary Table 3) and other electrodes. Neither were there any differences between the frontal ROIs concerning functional connectivity as a function of distance (Supplementary Fig. 2). Only the seed analysis revealed connectivity alterations involving frontal hubs, namely, between the right frontal ROI and contralateral frontal regions. This pattern remained after FDR correction (Supplementary Fig. 3).

### Classification analysis

We performed classification analyses to compare the discrimination power of our CNVs and the NPVs between samples. For bvFTD relative to controls, NPVs yielded an acceptable classification rate (83.8%). CNVs also yielded an acceptable classification rate (72.7%), although their power was lower than that of NPVs (Hotelling’s T-square test for sensitivity and specificity variables, *p*-value < 0.01). However, when NPVs were complemented by CNVs as joint predictors, their classification rate (87.4%) superseded the one obtained by NPVs alone (Hotelling’s T-square test for sensitivity and specificity variables, *p*-value < 0.01). For these classification results, we also analyzed the contribution of each variable from each category (NPVs and CNVs). The CNVs that contributed the most to the classification rate were the four ones associated with the seed analysis. The NPVs yielding greater classification accuracy were the abstraction capacity subtest from IFS, total IFS score, verbal fluency, and total ACE score (see Supplementary Table 4).

As regards the classification of AD and controls, NPVs also yielded acceptable rate (88.3%). Instead, the rate obtained through CNVs was below chance (44.9%). In addition, the combination of both variables yielded a rate similar to that obtained via NPVs alone (88.0%). The variables that contributed the most to the classification within the CNVs belonged to the seed analysis (connectivity values between left frontal and right temporal, and right frontal and left parietal regions). The NPVs that most contributed to classification were total IFS score, the verbal inhibitory control subtest from the IFS, total ACE score, and memory from ACE test (see Supplementary Table 5).

**Figure 4:**
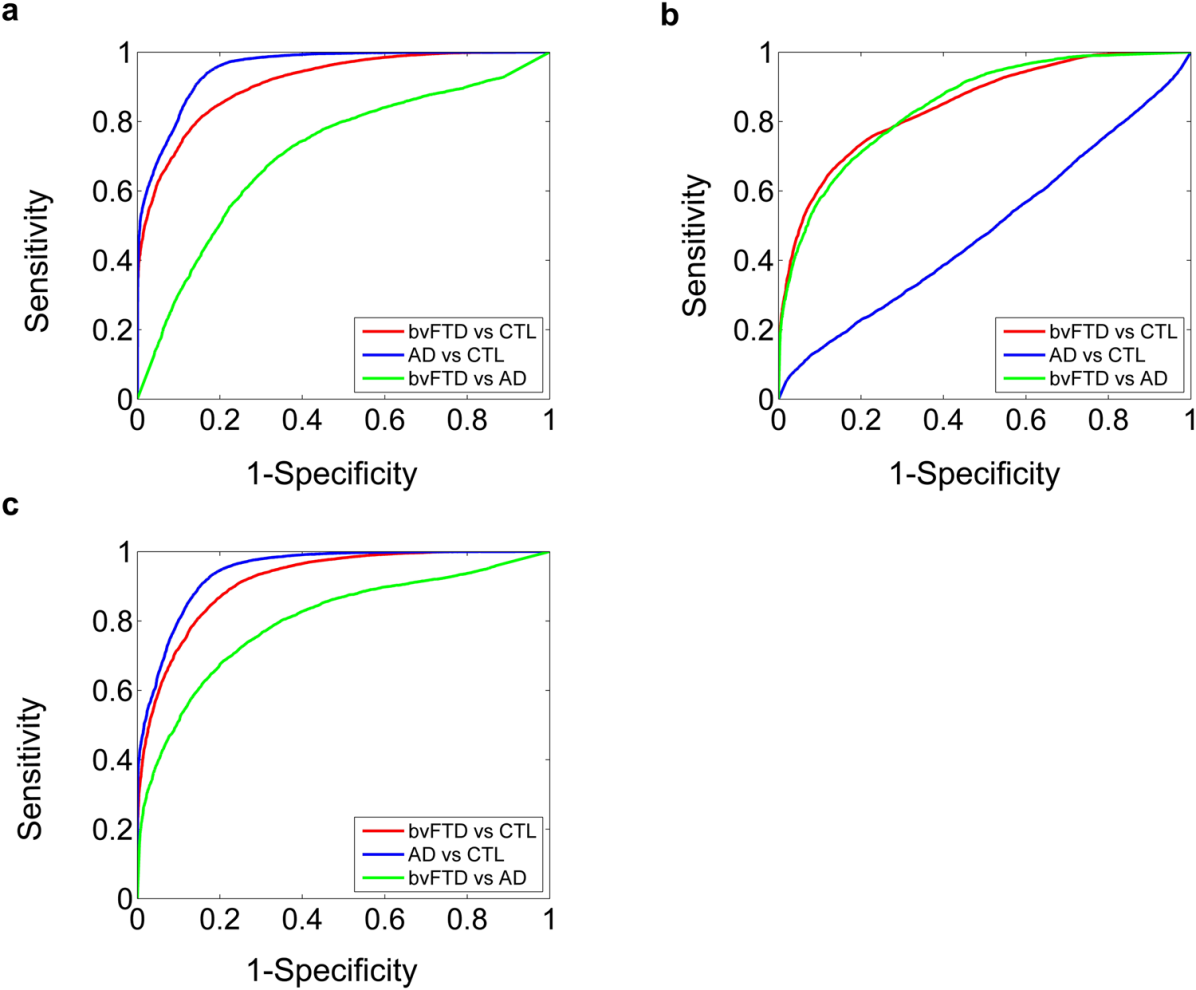
ROC curves for classification analyses. We calculated ROC curves for each classification with their corresponding groups of variables. Then the AUC were calculated to evaluate classification power. The AUC values yielded similar classification rates as those obtained in the other classification analyses. (**A**) ROC curves for classifications using neuropsychological variables. AUC values: bvFTD patients vs. controls = 0.76, AD patients vs. controls = 0.77, and bvFTD patients vs. AD patients = 0.65. (**B**) ROC curves for classifications using connectivity variables. AUC values: bvFTD patients vs. controls = 0.73, AD patients vs. controls = 0.54 and bvFTD patients vs. AD patients = 0.73. (**C**) ROC curves for classification using both neuropsychological and connectivity variables. AUC values: bvFTD patients vs. controls = 0.78, AD patients vs. controls = 0.77, and bvFTD patients vs. AD patients = 0.70.

Finally, the performance of NPVs to classify between bvFTD and AD patients (66.7%) was poorer than that offered by CNVs (72.9%) (Hotelling’s T-square test for sensitivity and specificity variables, *p*-value < 0.01). The combination of both types of variable did not enhance discrimination power between bvFTD and AD (72.9%). The CNVs with the higher contribution to the classification rates belonged to the seed and distance analyses (connectivity values between right frontal and left parietal regions, left frontal and right temporal areas, and connectivity distance from the right frontal ROI). The NPVs with the highest contribution were delay recall subtest from RAVLT test, the spatial working memory subtest from IFS test, and the verbal inhibitory control subtest from IFS test (see Supplementary Table 6). All sensitivity and specificity values are shown in Fig. 2 and in Supplementary Table 7. In addition, we have included ROC curves and estimated their AUC values (which reproduce the same classification rates reported above) to illustrate the classification performance in the Fig. 4.

## Discussion

This study aimed to identify whether information sharing indexed by EEG connectivity could reveal disease-specific alterations in bvFTD. Based on a novel connectivity measure (wSMI) and a multidimensional approach, we found reduced information sharing mainly across mid- and long-range frontotemporal hubs in bvFTD compared to controls. Moreover, these signatures were not observed in AD. Finally, such connectivity results enhanced classification of bvFTD patients from controls based on neuropsychological data, and they offered the best discrimination between bvFTD and AD patients. These findings suggest that EEG-derived connectivity analyses could offer important contributions to the ongoing quest of massively available, inexpensive biomarkers for bvFTD.

Relative to controls, bvFTD patients exhibited hypoconnectivity between frontal and temporal ROIs. This finding was further supported by our seed analysis, which revealed that frontal regions featured decreased connectivity with right temporal and left parietal locations. Although our analyses were not biased by the selection of a priori ROIs, the pattern of alteration that we found is consistent with the physiopathology of bvFTD^1^, characterized by progressive degeneration of fronto-temporal areas, such as the orbitofrontal cortex, the anterior cingulate, the insula, and medial temporal regions^27, 38^. Indeed, fMRI connectivity research has also shown hypoconnectivity between fronto-temporo and fronto-parietal regions^24, 44–46^), and within frontal areas (as shown by decreased connectivity in the Salience Network^47–49^). Our results align with such findings, further suggesting that early-stage atrophy in bvFTD may disrupt the exchange of signals between frontal and temporal hubs.

So far, only a few studies have assessed connectivity patterns in bvFTD with electromagnetic techniques, showing alterations in frontal connections. Key findings include reduced fronto-temporal connectivity was observed in alpha and beta frequencies^16^, disruption of frontal connectivity clusters in the alpha band^15^, and higher degree correlation compared to controls (and, thus, a more ordered network structure, suggesting suboptimal balance of small-world network architecture)^17^. Despite their different methodological approaches, these works converge in demonstrating frontal alterations in bvFTD via EEG-based connectivity analyses. To our knowledge, only one EEG study failed to find global and regional connectivity differences between bvFTD patients and healthy participants^13^. Based on the specificity of topographic analyses and the novel measure used, our study may present advantages to reveal connectivity alterations. In this sense, the novel information sharing measure we applied here (wSMI)^18, 20^ may hold considerable promise, as it captures non-linear coupling patterns while reducing spurious connectivity of common sources and controlling for the problem of volumetric conductance. Indeed, arguably because of these features, wSMI seems more sensitive than other widely used connectivity metrics^18^.

Distance analysis extended our results showing that reduced information sharing in bvFTD was driven by abnormalities in mid- and long-range frontal connections, with a relative preservation of short distance connections. Interestingly, this pattern of long-range abnormalities was also observed in a previous connectivity study in bvFTD patients^36^. Also, fMRI-based analysis of path length in bvFTD^44^ via graph theory has revealed alterations in functional integration across the whole brain, suggesting that long-range connections may be disrupted. Taken together, these findings and our present results point to decreased mid- and long-range (frontotemporal) connectivity as a potential hallmark of bvFTD patient.

These functional connectivity alterations in bvFTD were replicated with a low-density EEG setting (See Fig. 3). Although it only involves 20 electrodes (considerably fewer than the 128 used in our original analysis), this setting yielded the same patterns observed in our main study: frontal hypoconnectivity, decreased connectivity as a function of distance, and abnormal connections involving fronto-temporal and fronto-parietal regions. This indicates that our approach, based on the analysis of EEG functional connectivity via wSMI, might be successfully implemented with portable equipment featuring only a few electrodes. Moreover, we applied a very simple preprocessing pipeline (see Supplementary Data 4.) in which the quality of the signal does not depend of the number of channels acquired (e.g., to preserve as much of the original signal as possible, no ICA decomposition was performed). Although further research and replication studies are needed, these findings highlight that EEG (and even more the portable ones) might offer a promising method for massive application.

Our findings also highlight the sensitivity of a specific frequency range to reveal such patterns. Given the Tau parameters we employed for wSMI analysis, our results reflect connectivity alterations at a range of 8–20 Hz. This aligns with previous reports on bvFTD showing abnormal frontal connectivity in the alpha and beta frequency bands^15–17^. Such frequency ranges have also yielded significant alterations in bvFTD through power analyses focused on global measures^21^, specific fronto-temporal activity^23^, and frontal microstates^22^. The consistency of these patterns in bvFTD and their relation with specific fronto-temporal areas targeted by the disorder’s physiopathology^7, 38, 50^ suggest that disruptions of information sharing at 8–20 Hz may play an important role in this disease. Future works should be directly aimed to examine the specificity and sensitivity of alpha and beta dysfunctions in bvFTD.

Of note, results from our contrastive patient sample, composed of AD patients, suggest that the above patterns may be specific to bvFTD as opposed to neurodegeneration at large. Indeed, compared to controls, the AD group presented no connectivity alterations between fronto-temporal and fronto-parietal areas, and they evinced no connectivity decay in terms of distance. Frontal preservation is expected in AD patients, especially in early stages, given that their atrophy pattern mainly affects temporal and parietal regions^51^. Unlike frontal regions, these posterior areas are also more atrophied in AD than in bvFTD^52, 53^. The distinctiveness of the alterations we observed in bvFTD is underscored by previous EEG and fMRI studies on AD showing preserved frontal connectivity^54^ despite abnormal posterior connectivity^55–57^. Moreover, EEG studies directly comparing AD and bvFTD patients have reported differential connectivity alterations of posterior areas for the former and of frontal regions for the latter^15^. Thus, altered frontal network connectivity may constitute a critical feature distinguishing bvFTD from other neurodegenerative conditions.

The relevance of altered fronto-temporal connectivity^1^ for bvFTD is further highlighted by our classification analyses. Performance on neuropsychological tests allowed classifying bvFTD from controls at an acceptable rate (83.8%), which was better than that obtained through connectivity measures (72.7%). This is not surprising, given that the tests considered were designed to evaluate cognitive domains directly targeted by bvFTD^31, 58^. However, when neuropsychological measures were complemented by connectivity results, classification power improved considerably (87.4%). Furthermore, in classifying between bvFTD and AD patients, connectivity patterns yielded a discrimination rate (72.2%) above that obtained through neuropsychological measures alone (66.7%) and similar to the one yielded by the combination of both (72.9%).

Neuroimaging studies have also provided high classification rates in FTD and AD samples. An accuracy of 100% was reported in classifying between both AD and FTD patients from healthy controls^59^. Also, rates of 88%^60^ and 84.3%^59^ were obtained in discriminating between AD and bvFTD. Moreover, similar performance was reported in a resting-state functional connectivity study (92% of accuracy comparing bvFTD, AD and controls^48^), further highlighting the relevance of this brain property to identify bvFTD patients. Despite these promising results, the methods employed in these studies (e.g., SPECT, MRI, fMRI) present intrinsic limitations that restrict their massive implementation in clinical populations, such as their high cost, their low portability, and the difficulties for elderly subjects to complete imaging protocols due to claustrophobia, physical pain, movement limitations, presence of cardiac devices, or other medical issues.

Promisingly, EEG may represent a more affordable method to develop a biomarker for dementias. Although no previous EEG connectivity studies have performed classification analysis, research based on power spectral measures have reported varied classification rates. For bvFTD and controls classification, while some works shown higher classification rates^10^ (95% of accuracy), others shown middle^21^ (79% of accuracy), to even low levels of classification (50% accuracy for FTD classification^9^). For bvFTD and AD classification a rate of 71% was reported in ref. 21. Besides, they presented methodological limitations as the implementation of analysis that does not consider specific topographical differences^21^ (global field potential), or data-driven approaches^10^ where a few variables were selected for classification analysis from a vast set (over 30 considering different regions and frequency bands), which might undermine the reproducibility of the results.

Only two studies combined brain markers with neuropsychological measures to discriminate dementias. First, an EEG study^21^ showed that bvFTD patients could be distinguished from controls and AD patients with rates of 79% and 71%, respectively. In line with our findings, classification rates improved when neuropsychological variables were added. In addition, a resting-state fMRI study also showed a high classification rate (95%) between bvFTD and controls combining graph-theory metrics and social and executive function variables^24^. These findings highlight the relevance of combining neuropsychological assessment and brain markers to better characterize bvFTD patients. In particular, the use of EEG proves particularly promising for massive application due to its low cost, portability, and adaptability to patients with physical alterations, reduced mobility, or even counter-indications for neuroimaging analysis.

Admittedly, the above conclusions must be considered tentative on account of our study’s limitations. First, our sample sizes were modest, though not smaller than those reported in previous studies^13, 16^. In addition, the sample size analysis showed that the number of participants in our main findings was adequate to have a large effect size (as the ones obtained in our analyses, Cohen’s *d* > 0.80) and power. Finally, our findings were consistent across analyses and even when all analyses were repeated with a set of only 20 electrodes. Although further research with higher sample sizes is needed, these considerations suggest that our findings were not biased by the number of participants in each group. Also, on account of the sociodemographic differences between our bvFTD and AD samples, the analysis of each pathology relied on separate control groups. Thus, or claims for specificity depend on an indirect comparison. However, we found that age did not influence the classification based on connectivity metrics (see Supplementary Fig. 4 and Supplementary Fig. 5), and that such a variable yielded low classification rates on its own (see Supplementary Fig. 6). These two limitations should be circumvented in future research to test the robustness of our findings. Moreover, it would be critical to test whether similar results are obtained through portable equipments with fewer channels, as this would dramatically increase the chances of massive implementation of our method.

In sum, bvFTD is difficult to diagnose via conventional neuropsychological or routine neuroimaging evaluations, given its relatively early age of onset, its clinical overlap with other pathologies, and the variability of its underlying atrophy pattern^1, 2^. It is thus crucial to develop new tools to complement the current clinical toolkit and improve early diagnosis, assess the impact of therapy and, hence, alleviate the associated financial burdens. Here, through a combination of advanced approaches to EEG data, we showed that analyses of frontotemporal connectivity in specific frequency ranges could offer sensitive markers of bvFTD and enhance the classification rates obtained through neuropsychological measures. Although much more research is needed, these findings highlight the potential role of EEG measures in the ongoing quest for affordable and massively applicable frameworks to establish biomarkers of bvFTD.

## Electronic supplementary material

Supplementary information.
