# Supplementary material for: Towards affordable biomarkers of frontotemporal dementia: A classification study via network’s information sharing
Source: Sci Rep. 2017 Jun 19;7:3822. doi: 10.1038/s41598-017-04204-8 (PMC5476568; doi:10.1038/s41598-017-04204-8)
Supplement: Supplementary file 1 — Supplementary information. [file 41598_2017_4204_MOESM1_ESM.pdf]

Supplementary Data: Towards affordable biomarkers of frontotemporal dementia: A classification study via network's information sharing

Prof. Martin Dottori[1,2], Prof. Lucas Sedeño[1,2], Prof. Miguel Martorell Caro[1], Prof. Florencia Alifano[1], Prof. Eugenia Hesse[1,2], Prof. Ezequiel Mikulan[1,2], Ph.D. Adolfo M. García[1,2,3], Prof. Amparo Ruiz-Tagle[4], Ph.D. Patricia Lillo[5,6], Ph.D. Andrea Slachevsky[6,7,8,9,10], Ph.D. Cecilia Serrano[11], Ph.D. Daniel Fraiman[2,12], Ph.D. Agustin Ibanez[2,13,14,15,16,17*]

Authors' affiliations

1 Laboratory of Experimental Psychology and Neuroscience (LPEN), Institute of Cognitive and Translational Neuroscience (INCyT), INECO Foundation, Favaloro University, Buenos Aires, Argentina

2 National Scientific and Technical Research Council (CONICET), Buenos Aires, Argentina

3 Faculty of Education, National University of Cuyo (UNCuyo), Mendoza, Argentina

4 Centre for Advanced Research in Education, Periodista Jose Carrasco, Santiago, Chile

5 Departamento de Neurologia Sur, Facultad de Medicina, Universidad de Chile, Santiago, Chile

6 Gerosciences Center for Brain Health and Metabolism, Santiago, Chile

7 Physiopathology Department, ICBM and East Neuroscience Department, Faculty of Medicine, University of Chile, Santiago, Chile

8 Cognitive Neurology and Dementia, Neurology Department, Hospital del Salvador, Santiago, Chile

9 Centre for Advanced Research in Education, Santiago, Chile

10 Servicio de Neurología, Departamento de Medicina, Clínica Alemana-Universidad del Desarrollo, Santiago, Chile

11 Memory and Balance Clinic, Buenos Aires, Argentina

12 Laboratorio de Investigación en Neurociencia, Universidad de San Andrés, Buenos Aires, Argentina

13 Neuroscience Research Australia, Sydney, Australia and School of Medical Sciences, The University of New South Wales, Sydney, Australia

14 Australian Research Council (ACR) Centre of Excellence in Cognition and its Disorders, Macquarie University, New South Wales, Australia

15 Institute of Cognitive and Translational Neuroscience (INCyT), INECO Foundation, Favaloro University, Buenos Aires, Argentina

16 Universidad Autónoma del Caribe, Barranquilla, Colombia

17 Center for Social and Cognitive Neuroscience (CSCN), School of Psychology, Universidad Adolfo Ibáñez, Santiago de Chile, Chile

* **Corresponding author:** Agustin Ibanez

Corresponding author's address: Pacheco de Melo 1860, C1126AAB, Buenos Aires, Argentina

Corresponding author's phone and fax: +54 (11) 4807-4748

Corresponding author's e-mail address: aibanez@ineco.org.ar

1. **Supplementary Data 1. Patient recruitment details.**
2. **Supplementary Data 2. Sample size estimation.**
3. **Supplementary Data 3. Neuropsychological tests details.**
4. **Supplementary Data 4. EEG Preprocessing.**
5. **Supplementary Data 5. Weighted symbolic mutual information (wSMI) details.**
6. **Supplementary Data 6. Calculation details of functional connectivity by distance function.**
7. **Supplementary Data 7. Support Vector Machine algorithm.**
8. **Supplementary Data 8.Classification parameters for SVM training phase.**
9. **Supplementary Data 9. Classification of patients by the age variable.**
10. **Supplementary Tables.**
11. **Supplementary Figures.**

Supplementary Data 1. Patient recruitment details.

Thirteen patients were recruited who met the revised criteria for probable behavioral variant frontotemporal dementia (bvFTD)[1]. Diagnosis was made by a panel of experts in dementia, including cognitive neurologists, psychiatrists, and neuropsychologists, as in previous works of our group[2,3]. As part of a standard clinical examination, all patients completed a battery of neuropsychological, psychiatric, and neurological exams as well as a magnetic resonance imaging (MRI) protocol. The patients presented prominent changes in their personality and social behavior, alongside functional impairment, an observation verified by caregivers at the initial evaluation. All patients exhibited frontal or temporal atrophy on MRI.

Thirteen patients with Alzheimer's disease (AD) were assessed following the same institute's standard clinical examination protocol detailed before. This sample was included to evaluate the specificity of potential alterations in bvFTD patients by comparing connectivity abnormalities in each condition. All patients were in early/mild stages of dementia. Those who primarily presented with language deficits were excluded.

Supplementary Data 2. Sample size estimation.

To estimate the appropriate sample size for our main analyses, we have performed an estimation analysis in G*Power 3.1 –a statistical software widely used in social and behavioral research[4]. Given our statistical design (Wilcoxon test for different variables comparing two groups), we considered the following parameters: (i) effect size Cohen's *d* = 0.9 (this large effect was selected based on a set of previous studies in the area[5]), (ii) alpha level of *p* = .05, and (iii) power = 0.8 (higher than the median of powers analyzed in[5] for detecting large effect sizes). This analysis showed that a sample size of 13 and 25 for the groups (considering the relation between samples similar to those used in this work, N1/N2 = 1.92) is adequate to detect the estimated effects.

Supplementary Data 3. Neuropsychological tests details.

The INECO Frontal Screening battery[6] is a sensitive tool to detect executive dysfunction in patients with dementia[7]. This test includes the following eight subtests: (1) motor programming (Luria series, "fist, edge, palm"); (2) conflicting instructions (in which subjects are asked to hit the table once when the administrator hit it twice or to hit the table twice when the administrator hit it only once); (3) motor inhibitory control; (4) numerical working memory (backward digit span); (5) verbal working memory (months backwards); (6) spatial working memory (a modified Corsi tapping test); (7) abstraction capacity (inferring the meaning of proverbs); and (8) verbal inhibitory control (a modified Hayling test). The global score of the IFS (the sum of the subtests, with a maximum value of 30) was also considered, as in previous works [8].

The Addenbrooke's Cognitive Examination[9] is a sensitive tool to detect early stages of dementiaand, more particularly,to distinguish between AD and FTD patients[10].This test evaluates orientation, attention, memory, verbal fluency, language, and visuospatial ability (with a maximum total score of 100).

The Rey Auditory Verbal Test is measure based on verbal memory that provides scores for distinct features/characteristics of memory. The test consist in the auditory presentation and the recalled of a list of

words (A), the presentation of another list of words of distractor character, and the evaluation of immediate and delayed recalls of List A, as well as recognition testing. This test was proved to be relevant in distinguishing patients with bvFTD and AD patients[11].

Supplementary Data 4. EEG preprocessing.

EEG data was down-sampled to 512 Hz and high-pass filtered at 0.5 HZ with EEGLAB software. Segments of data containing excessive noise, eye movement or muscular artifacts were rejected by visual examination. For every subject, a minimum of 300 seconds of cleaned data were used for connectivity calculation. In order to preserve as mucho of the original signal as possible, no ICA decomposition was performed. The noisy channels were identified by calculating their normalized standard deviation (z-values of the standard deviation above 5 where used as threshold criteria) and then manually rejected or retained through visual confirmation. Rejected channels were interpolated using spherical spline interpolation.

Supplementary Data 5. Details of the weighted symbolic mutual information (wSMI) analysis.

The weighted Symbolic Mutual Information (wSMI) measure[12] is based on the estimation of a non-linear index of information sharing between two signals. K samples of the signal separated by a time tau are taken into account and define a series of symbols based on the order relation between the magnitudes of samples. These symbols represent the temporal evolution of the signals. The measure is calculated for each pair of electrodes in segments of the signal (defined with a length of 1,000 ms) based on the marginal probability distribution functions and the joint probability density function estimated for the series of symbols obtained for the entire signals:

$$(1) \qquad \omega SMI(\hat{X},\hat{Y}) = \frac{1}{\log(k!)} \sum_{\hat{x}\epsilon\hat{X}} \sum_{\hat{y}\epsilon\hat{Y}} \omega(\hat{x},\hat{y}) p(\hat{x},\hat{y}) \log\frac{p(\hat{x},\hat{y})}{p(\hat{x})p(\hat{y})}$$

In formula (1) above, $\hat{x}$ and $\hat{y}$ represent the different classes of possible symbols in the two signals to be compared, $p(\hat{x})$ is the probability of occurrence of symbol $\hat{x}$, and$p(\hat{x},\hat{y})$ is the joint probability of occurrence for corresponding symbols. The factor $\omega(\hat{x},\hat{y})$ modulates the contribution of the different pairs of symbols to the measure. Their values are equal to zero when $\hat{x}$=$\hat{y}$ and $\hat{x}$ =-$\hat{y}$, and is equal to 1 in the other cases. The application of these weights avoids emphasizing pairs of symbols which may be coupled due to common-source artifacts.

Given the interest of assessing alpha and beta bands[13-15], the wSMI parameters were set on tau=16 (sensitive to frequency range of 8-20 Hz) and K=3.

Supplementary Data 6. Calculation details of functional connectivity by distance function.

The correlation function is defined as the average correlation between electrodes separated by distance *d*[2]. The connectivity values of the connections of each ROI were ordered by distance and grouped every 100 connections; then, each of these groups of connections was averaged to obtain the mean connectivity through all distances. This mean connectivity as a function of distance was calculated for each subject's group of connections. The level of connectivity decay indicates whether connectivity of a ROI is affected in a patient group regarding controls. Here, we adapted a previously reported analysis protocol[2] and computed the correlation function excluding connections between electrodes within a ROI.

Supplementary Data 7. Support vector machine algorithm.

The support vector machine (SVM) used is a binary classifier capable of finding the best hyperplane separating data variables according their class[16]. The vectors of variables used can be mapped to a higher dimensional space through a special kernel functions. In particular, we used a linear classification using the dot product as a kernel function.

Given a set of d variables, $\boldsymbol{X_i}$ vectors are defined containing the data of these variables associated to each subject, with $\boldsymbol{Y_i}$ scalars representing binary categories to classify.

The algorithm finds a hyperplane $F(\mathbf{X}) = \mathbf{X}.\boldsymbol{\beta} + C = 0$ that separates the vectors $\boldsymbol{X_i}$ with different $\boldsymbol{Y_i}$ values (categories) and minimizes the distances from it relative to each vector $\boldsymbol{X_i}(|\boldsymbol{\beta}.\boldsymbol{X_i}+C|)$.

Where the data are not completely separable, SVM finds a hyperplane that separates many, but not all data points. This is achieved by penalizing cases of vectors which do not agree with the binary separation determined by the hyperplane.

Supplementary Data 8. Classification parameters for the SVM training phase.

The SVM algorithm involved a training phase and a classification phase. In the training phase, a subset of the data (corresponding to seven randomly selected subjects from each group) and their corresponding classes (subject condition) were used to determine the classification parameters. Relative to the total number of subjects in each comparison, the subsets of subjects considered for the training phase represented 37% for bvFTD vs. controls, 45% for AD vs. controls, and 54% for bvFTD vs. AD. These percentages of data for training phase are similar to those used in previous studies(e.g.,[17]).

Supplementary Tables.

Supplementary Table 1. Neuropsychological and connectivity variables used for the classification

<table>
<tr><td colspan="2">NPVs</td><td>CNVs</td></tr>
<tr><td>Instrument</td><td>Scores</td><td>Metrics</td></tr>
<tr><td>Addenbrooke's Cognitive Examination</td><td>Total Score
Attention
Memory
Verbal Fluency
Language
Visuospatial Abilities</td><td rowspan="3">Connectivity-Distance Right Frontal
Connectivity-Distance Left Frontal
Seed Right Frontal-Right Temporal
Seed Right Frontal-Left Parietal
Seed Left Frontal-Right Temporal
Seed Left Frontal-Left Parietal</td></tr>
<tr><td>Rey Auditory Verbal Learning Test</td><td>Immediate
Distractor
Delay
False Positive</td></tr>
<tr><td>INECO Frontal Screening</td><td>Total Score
Motor programming
Motor Inhibitory Control
Conflicting Instructions
Backward Digit Span
Verbal Working Memory
Spatial Working Memory
Abstraction Capacity
Verbal Inhibitory Control</td></tr>
</table>

Supplementary Table 2. Connectivity mean values comparison between ROIs (bvFTD vs controls)

DFT vs CTL	R1	R2	R3	R4	R5	R6	R7
R1	-	.45	.09	.74	.47	.04	.45
R2	-	-	.02	.57	.45	.11	.45
R3	-	-	-	.57	.57	.10	.53
R4	-	-	-	-	.70	.70	.45
R5	-	-	-	-	-	.53	.57
R6	-	-	-	-	-	-	.57
R7	-	-	-	-	-	-	-

R1 = left frontal, R2 =right frontal, R3 = right temporal, R4=right posterior, R5= left posterior, R6=left temporal and R7= Central. *P*-values obtained with Wilcoxon test comparison (corrected by FDR) for the connectivity mean values between ROIs. bvFTD vs control group comparison. ROIs used: left frontal (R4), right frontal (R3), left temporal (R5), right temporal (R2), left posterior (R6), right posterior (R1) and Central (R7).

Supplementary Table 3. Connectivity mean values comparison between ROIs (AD vs controls).

AD/CTL	R1	R2	R3	R4	R5	R6	R7
R1	-	.67	.27	.92	.98	.98	.53
R2	-	-	.48	.59	1.0	.30	.27

R1 = left frontal, R2 =right frontal, R3 = right temporal, R4=right posterior, R5= left posterior, R6=left temporal and R7= Central. *P*-values obtained with Wilcoxon test comparison (uncorrected) for the connectivity mean values between ROIs. AD vs control group comparison. ROIs used: left frontal (R1), right frontal (R2), left temporal (R6), right temporal (R3), left posterior (R5), right posterior (R4) and Central (R7).

Supplementary Table 4. Z-scores of accuracy rates for bvFTD vs controls

bvFTD vs Controls			
Variable Name	Z-score	Variable Name	Z-score
Abstraction capacity (IFS)	1.40	Seed right frontal-left parietal	0.94
Verbal fluency (ACE)	1.39	Seed left frontal-right temporal	0.71
Total score (ACE)	1.19	Seed right frontal-right temporal	0.69
Immediate recall (RAVLT)	0.84	Seed left frontal-left parietal	0.13
Total score (IFS)	0.81	Connectivity-distance right frontal	-1.04
Memory (ACE)	0.47	Connectivity-distance left frontal	-1.43
False positive (RAVLT)	0.46		
Delayed recall (RAVLT)	0.37		
Visuospatial abilities (ACE)	0.23		
Distractor (RAVLT)	0.21		
Language (ACE)	0.02		
Verbal inhibitory control (IFS)	0.02		
Conflicting instructions (IFS)	-0.20		
Spatial working memory (IFS)	-0.23		
Attention (ACE)	-0.71		
Motor programming (IFS)	-1.24		
Verbal working memory (IFS)	-1.55		
Motor inhibitory control (IFS)	-1.55		
Backward digit span (IFS)	-1.93		

Supplementary Table 4. Z-scores of accuracy rates for bvFTD vs controls. In this table the z-scores of accuracy rates of classification related to individual variables respect to each group of variables are shown. The accuracy rates of classification were calculated for a bvFTD vs controls classification where only one variable was used to implement the SVM algorithm. The z-score of the accuracy rates of classification gives an idea of the importance of each variable in this group of variables respect to the classification ($z(v_i) = (v_i - mean([v_1, v_{2,\ldots,} v_n])/(std([v_1, v_{2,\ldots,} v_n]))$, where v_i is the proportion of correct classification using solely the predictor variable i).

Supplementary Table 5. Z-scores of accuracy rates for AD vs controls

AD vs Controls			
Variable Name	Z-score	Variable Name	Z-score
Delayed recall (RAVLT)	1.25	Seed left frontal-left parietal	1.01
Verbal inhibitory control (IFS)	1.21	Seed right frontal-right temporal	0.90
Spatial working memory (IFS)	1.02	Seed right frontal-left parietal	0.71
Total score (IFS)	0.78	Connectivity-distance right frontal	-0.46
Memory (ACE)	0.76	Connectivity-distance left frontal	-0.84
Total score (ACE)	0.73	Seed left frontal-right temporal	-1.32
Verbal fluency (ACE)	0.69		
Immediate recall (RAVLT)	0.52		
Distractor (RAVLT)	0.24		
Attention (ACE)	0.21		
Abstraction capacity (IFS)	0.11		
Motor programming (IFS)	0.10		
Visuospatial abilities (ACE)	0.02		
Motor Inhibitory control (IFS)	-0.67		
Conflicting instructions (IFS)	-0.71		
Language (ACE)	-0.92		
Verbal working memory (IFS)	-1.73		
Backward digit span (IFS)	-1.73		
False positive (RAVLT)	-1.88		

Supplementary Table 5: Z-scores of accuracy rates for AD vs controls. In this table the z-scores of accuracy rates of classification related to individual variables respect to each group of variables are shown. The accuracy rates of classification were calculated for an AD vs controls classification where only one variable was used to implement the SVM algorithm. The z-score of the accuracy rates of classification gives an idea of the importance of each variable in this group of variables respect to the classification ($z(v_i) = (v_i\text{-mean}([v_{1,} v_{2,\ldots,} v_n])/(\text{std}([v_{1,} v_{2,\ldots,} v_n]))$).

Supplementary Table 6. Z-scores of accuracy rates for bvFTD vs AD

bvFTD vs AD			
Variable Name	Z-score	Variable Name	Z-score
Memory (ACE)	1.92	Seed right frontal-left parietal	1.93
Delayed recall (RAVLT)	1.56	Seed left frontal-right temporal	0.05
Abstraction capacity (IFS)	1.07	Connectivity-distance right frontal	-0.21
Spatial working memory (IFS)	0.84	Connectivity-distance left frontal	-0.33
Total score (ACE)	0.83	Seed right frontal-right temporal	-0.47
Attention (ACE)	0.54	Seed left frontal-left parietal	-0.96
Verbal inhibitory control (IFS)	0.52		
False positive (RAVLT)	0.49		
Motor programming (IFS)	0.30		
Immediate recall (RAVLT)	-0.03		
Total score (IFS)	-0.19		
Distractor (RAVLT)	-0.54		

Backward digit span (IFS)	-0.69		
Language (ACE)	-0.72		
Visuospatial abilities (ACE)	-0.97		
Motor inhibitory control (IFS)	-1.01		
Conflicting instructions (IFS)	-1.21		
Verbal fluency (ACE)	-1.29		
Verbal working memory (IFS)	-1.42		

Supplementary Table 6. Z-scores of accuracy rates for bvFTD vs AD. In this table the z-scores of accuracy rates of classification related to individual variables respect to each group of variables are shown. The accuracy rates of classification were calculated for a bvFTD vs AD classification where only one variable was used to implement the SVM algorithm. The z-score of the accuracy rates of classification gives an idea of the importance of each variable in this group of variables respect to the classification ($z(v_i) = (v_i\text{-mean}([v_1, v_{2,\ldots,}, v_n])/(\text{std}([v_1, v_{2,\ldots,}, v_n]))$).

Supplementary Table 7. Classification results of sensitivity and specificity for groups comparisons.

	NPVs		CNVs		NPVs + CNVs	
	Sens.	1 – Spec.	Sens.	1 - Spec.	Sens.	1 - Spec.
bvFTD vs Ctrls.	0.83 (0.14)	0.16 (0.12)	0.87 (0.18)	0.32 (0.15)	0.9 (0.12)	0.13 (0.1)
AD vs Ctrls	0.84 (0.12)	0.1 (0.08)	0.57 (0.27)	0.59 (0.18)	0.85 (0.12)	0.11 (0.08)
bvFTD vs AD	0.68 (0.2)	0.35 (0.19)	0.72 (0.2)	0.28 (0.21)	0.77 (0.18)	0.31 (0.2)

Means and (standard deviation).CNVs = Connectivity variables, NPVs = Neuropsychological variables, Sens. = Sensitivity, Spec. = Specificity, bvFTD = behavioral variant frontotemporal dementia, AD= Alzheimer's disease, Ctrl = controls.

Supplementary Figures

Supplementary Figure 1: Effect sizes for analyses of connectivity as function of distance in the comparison between bvFTD patients and controls

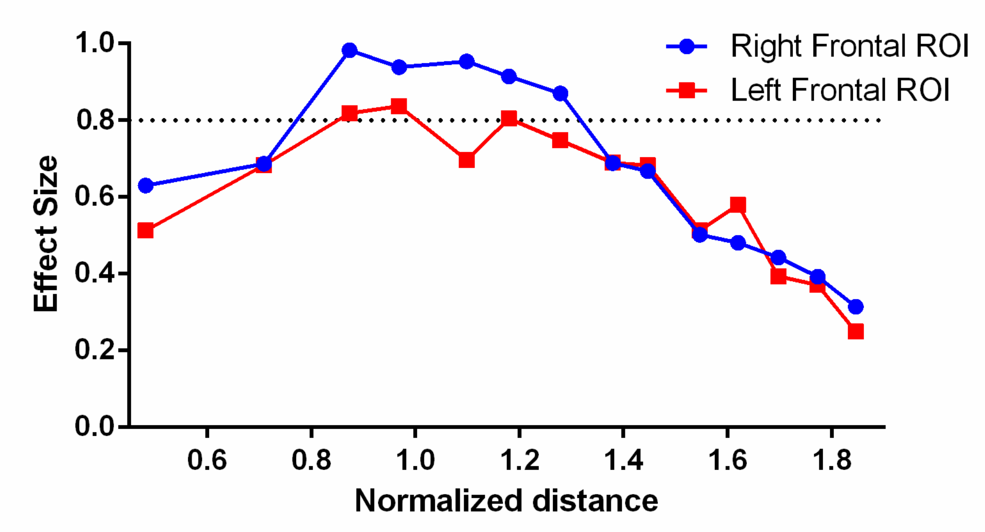

Supplementary Figure 1. Effect sizes for analyses of connectivity as function of distance in the comparison between bvFTD patients and controls. For a set of distances, we calculated the effect size (Cohen's *d)* of the connectivity in the comparison between bvFTD patients and controls. These effect sizes are shown for connectivity patterns associated to each frontal ROI yielding significant differences in the analyses of connectivity as a function of distance. The dashed line marks the limit of large effect sizes (Cohen's d = 0.8). The most important effect sizes appear in the middle range distance (0.8-1.4) while less effect size is observed for short range distance (0-0.8) and long range distance (1.4-1.8).

Supplementary Figure 2. Functional connectivity analysis (AD group).

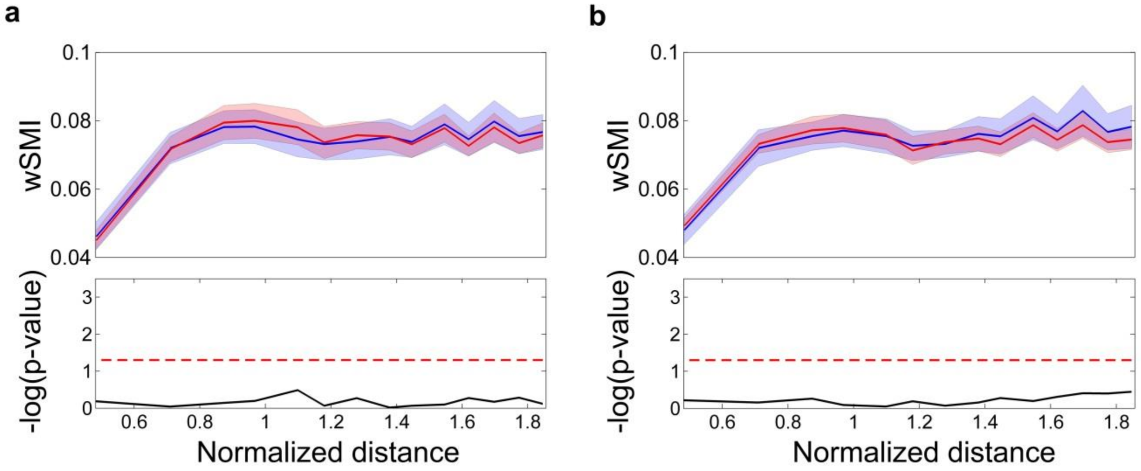

Supplementary Figure 2. Functional connectivity analysis (AD group). A. Connectivity as function of distance of the left frontal ROI: AD patients (red) and controls (blue). Results are shown as -log (*p*-value) by distance; *p*-values crossing the dotted line are < .05**. B. Connectivity as a function of distance of the Frontal Right ROI:** AD (red) and controls (blue). Results are shown as -log (*p*-value) by distance; *p*-values crossing the dotted line are < .05**.**

Supplementary Figure 3. Seed analysis for the comparison between AD and controls.

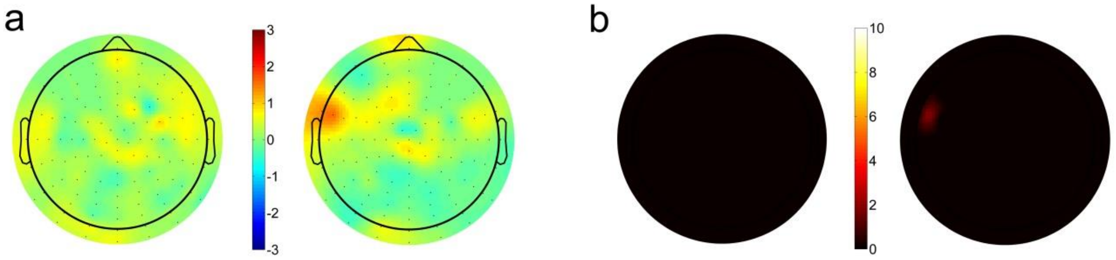

Supplementary Figure 3. Seed analysis for the comparison between AD and controls. A. Seed analysis (median values): scalp maps of the median value of *p*-values (from Wilcoxon test between AD and their controls) are shown for the left frontal (left) and right frontal (right) seeds. The color bar indicates -log [median (*p*-values)] times the sign of W, where W is the Wilcoxon statistics minus the expected value under the null hypothesis. Values > 1.3 or < -1.3 are statistically significant. **B. Seed analysis (FDR correction)**: scalp maps quantifying the number of connections (associated to the seed ROI) which yielded significant differences (*p*-value from Wilcoxon test between AD and controls with FDR < .05) for each electrode. The maps show the results for left frontal (left) and right frontal (right) seeds. The color bar indicates the number of connections with statistically significant differences (*p*-values< .05).

Supplementary Figure 4. Comparison of values of connectivity variables in the control samples, grouped by age.

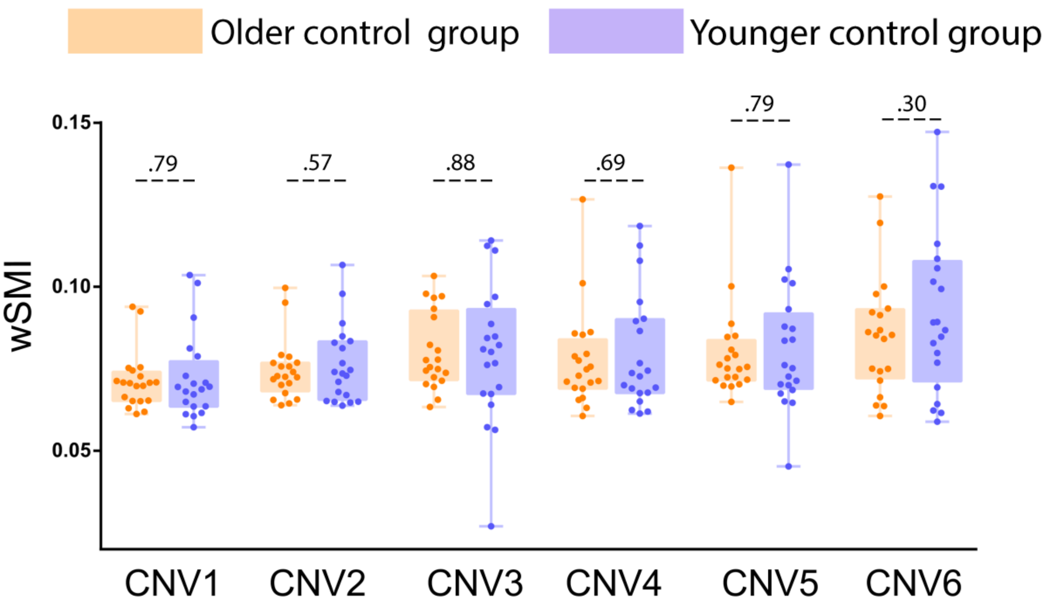

Supplementary Figure 4. Comparison of values of connectivity variables in controls sample grouped by age. To evaluate the effect of age on the connectivity variables used in the subjects classification, we

analyzed these metrics dividing the control group in two: Older Control Group (*n* = 20, *M*= 71.75, *SD* = 4.04) and a Younger Control Group (*n*=20, *M*= 58.1, *SD* = 5.97).T-tests were used to compare each connectivity measure between groups.CNV1: right frontal connectivity distance, CNV2: left frontal connectivity distance, CNV3: connectivity between the right frontal seed and right-temporal hubs, CNV4: connectivity between right frontal seed and left parietal hub, CNV5: connectivity between left frontal seed and right temporal hubs, CNV6: connectivity between left frontal seed and left parietal hubs. No significant differences were founded in any measure (*p*-values are showed above the dotted line).

Supplementary Figure 5. Comparison of values of connectivity variables in controls sample, grouped by age

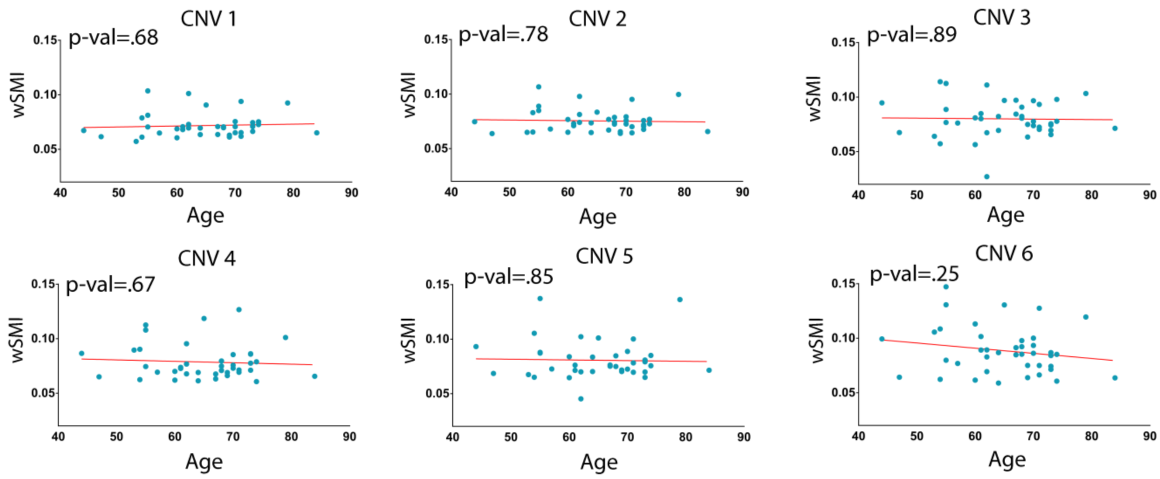

Supplementary Figure 5. Comparison of values of connectivity variables in controls sample grouped by age. To evaluate the effect of age on the connectivity variables used for classification, we also implemented a linear regression analysis. The variables analyzed were CNV1: right frontal connectivity distance, CNV2: left frontal connectivity distance, CNV3: connectivity between the right frontal seed and right-temporal hubs, CNV4: connectivity between right frontal seed and left parietal hub, CNV5: connectivity between left frontal seed and right temporal hubs, CNV6: connectivity between left frontal seed and left parietal hubs. No significant association was founded (*p*-values and β parameters of linear regression adjusts are showed in each panel).

Supplementary Figure 6. Classification of subjects with the age variable

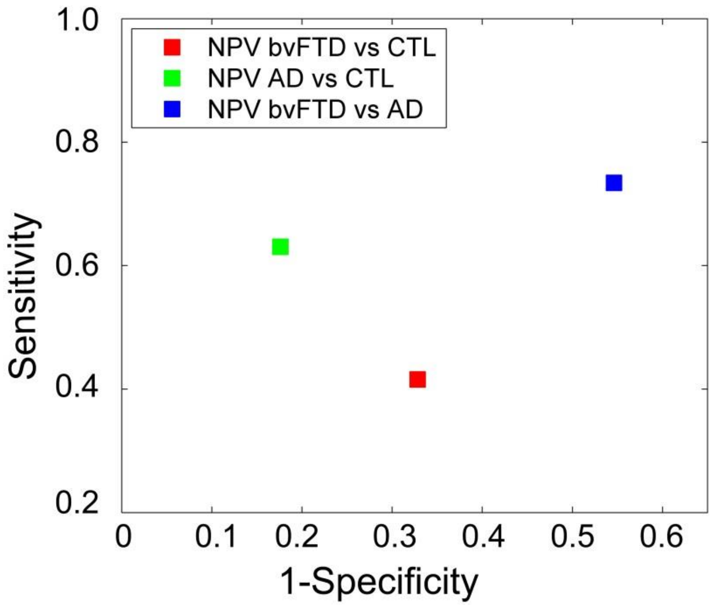

Supplementary Figure 6. Classification of subjects with the age variable. Using a SVM classification based on the age variable, we tested the irrelevance in the classification. We expected that, if age can explain the power classification obtained when we used connectivity or neuropsychological variables, then the classification rate given only by age should be better or, at least, similar than the obtained in the other classification analyses. We obtained a classification rate of 60% for bvFTD respect to Controls analyses, a 77% for AD respect to Controls and a 59% for bvFTD respect to AD. The sensitivity and 1 – specificity and their standard deviations were respectively 0.42 (0.22) and 0.33 (0.22) for bvFTD respect to controls, 0.62 (0.05) and 0.17(0.27) for AD respect to controls and 0.73(0.12) and 0.55(0.16) for bvFTD respect to AD.

1 Rascovsky, K. *et al.* Sensitivity of revised diagnostic criteria for the behavioural variant of frontotemporal dementia. *Brain : a journal of neurology* **134**, 2456-2477, doi:10.1093/brain/awr179 (2011).

2 Garcia-Cordero, I. *et al.* Stroke and Neurodegeneration Induce Different Connectivity Aberrations in the Insula. *Stroke; a journal of cerebral circulation* **46**, 2673-2677, doi:10.1161/STROKEAHA.115.009598 (2015).

3 Baez, S. *et al.* Comparing moral judgments of patients with frontotemporal dementia and frontal stroke. *JAMA neurology* **71**, 1172-1176, doi:10.1001/jamaneurol.2014.347 (2014).

4 Faul, F., Erdfelder, E., Lang, A. G. & Buchner, A. G*Power 3: a flexible statistical power analysis program for the social, behavioral, and biomedical sciences. *Behavior research methods* **39**, 175-191 (2007).

5 Szucs, D. & Ioannidis, J. P. Empirical assessment of published effect sizes and power in the recent cognitive neuroscience and psychology literature. *PLoS biology* **15**, e2000797, doi:10.1371/journal.pbio.2000797 (2017).

6 Torralva, T., Roca, M., Gleichgerrcht, E., Lopez, P. & Manes, F. INECO Frontal Screening (IFS): a brief, sensitive, and specific tool to assess executive functions in dementia. *Journal of the International Neuropsychological Society : JINS* **15**, 777-786, doi:10.1017/S1355617709990415 (2009).

7 Gleichgerrcht, E., Roca, M., Manes, F. & Torralva, T. Comparing the clinical usefulness of the Institute of Cognitive Neurology (INECO) Frontal Screening (IFS) and the Frontal Assessment Battery (FAB) in frontotemporal dementia. *Journal of clinical and experimental neuropsychology* **33**, 997-1004, doi:10.1080/13803395.2011.589375 (2011).

8 Ibanez, A. *et al.* The neural basis of decision-making and reward processing in adults with euthymic bipolar disorder or attention-deficit/hyperactivity disorder (ADHD). *PloS one* **7**, e37306, doi:10.1371/journal.pone.0037306 (2012).

9 Mathuranath, P. S., Nestor, P. J., Berrios, G. E., Rakowicz, W. & Hodges, J. R. A brief cognitive test battery to differentiate Alzheimer's disease and frontotemporal dementia. *Neurology* **55**, 1613-1620 (2000).

10 Hsieh, S., Schubert, S., Hoon, C., Mioshi, E. & Hodges, J. R. Validation of the Addenbrooke's Cognitive Examination III in frontotemporal dementia and Alzheimer's disease. *Dementia and geriatric cognitive disorders* **36**, 242-250, doi:10.1159/000351671 (2013).

11 Ricci, M., Graef, S., Blundo, C. & Miller, L. A. Using the Rey Auditory Verbal Learning Test (RAVLT) to differentiate alzheimer's dementia and behavioural variant fronto-temporal dementia. *The Clinical neuropsychologist* **26**, 926-941, doi:10.1080/13854046.2012.704073 (2012).

12 King, J. R. *et al.* Information sharing in the brain indexes consciousness in noncommunicative patients. *Current biology : CB* **23**, 1914-1919, doi:10.1016/j.cub.2013.07.075 (2013).

13 Lindau, M. *et al.* Quantitative EEG abnormalities and cognitive dysfunctions in frontotemporal dementia and Alzheimer's disease. *Dementia and geriatric cognitive disorders* **15**, 106-114, doi:67973 (2003).

14 Nishida, K. *et al.* Differences in quantitative EEG between frontotemporal dementia and Alzheimer's disease as revealed by LORETA. *Clinical neurophysiology : official journal of the International Federation of Clinical Neurophysiology* **122**, 1718-1725, doi:10.1016/j.clinph.2011.02.011 (2011).

15 Nishida, K. *et al.* EEG microstates associated with salience and frontoparietal networks in frontotemporal dementia, schizophrenia and Alzheimer's disease. *Clinical neurophysiology : official journal of the International Federation of Clinical Neurophysiology* **124**, 1106-1114, doi:10.1016/j.clinph.2013.01.005 (2013).

16 WS, N. What is a support vector machine? *Nature Biotechnology* **25**, 1565-1567 (2006).

17 Chandaka, S., Chatterjee, A. & Munshi, S. Cross-correlation aided support vector machine classifier for classification of EEG signals. *Expert Systems with Applications* **36**, 1329–1336, doi:10.1016/j.eswa.2007.11.017 (2009).
